# A “call to bear the burden of a long twilight struggle”: a special issue on social determinants of health assessment and intervention

**DOI:** 10.1093/jncics/pkae028

**Published:** 2024-04-24

**Authors:** Matthew F Hudson

**Affiliations:** Department of Medicine, Prisma Health Cancer Institute, Greenville, SC, USA

The Editor in Chief of *JNCI Cancer Spectrum* encouraged this journal and its audience to “bravely” undertake and disseminate cancer research (1). In most instances, there is no braver gesture than scrutinizing one’s self. Here, we invite our audience to critically consider our individual and collective role in tolerating or (un)intentionally sustaining a context portending cancer. More poignantly, our Journal invites us to scrutinize our individual and collective contribution to an infrastructure and interaction producing different care availability, processes, and outcomes disadvantaging some populations suffering from cancer. Collectively, these considerations are social determinants of health (SDOH) and cancer care (2). The field of cancer research persists in examining the role environment [eg, where we are born, grow, live, work, and age (3)] plays in cancer screening (4), incidence (5), care (6), and mortality (7). Yet, movement from discovery to intervention befuddles us. The sheer cooperative and interdependent nature of social determinants challenge us to clarify where and how to intervene. *JNCI Cancer Spectrum* encourages our field to critically examine the state of the science and ourselves regarding SDOH address.

First, it behooves cancer researchers and practitioners to distinguish between social determinants of health [or, particularly desirable, wellness (8)] and social determinants of care. Social determinants of health and wellness will require our attention to essential public health services (9). We particularly encourage the instruments clinical researchers and practitioners frequently under consider in health interventions—legal and regulatory action protecting and improving health and wellness. Social determinant address will require clinical investment beyond legislation advocacy; address requires our field to explicitly consider who and how legislators implement and, most critically, enforce laws equitably (10). Intervention design and implementation requires our clinical research enterprise to expand beyond the more familiar, yet equally daunting, health service enterprise requiring similar attention. Social determinants of care require keen attention to care facility location (11), care provision (12), care team constitution (13,14), and survivorship (15). Collective attention to social determinants of health and wellness and care requires a multilevel (16) research approach considering clinician, organization, community, and policy’s combined impact on patient and community outcomes.

Second, SDOH researchers should understand active opponents deem competencies and perspectives advantageous for SDOH and care exploration unnecessary (and disadvantageous). Opponents strongly disavow the notion that social determinants (eg, behavioral, economic, and environmental factors) exert a greater influence on patient health than clinicians or the clinical enterprise (17). Indeed, opponents challenge SDOH inquiry as an implicit medical fatalism (18) that compromises technological innovation (17). Consequently, SDOH researchers should prepare for resistance when undertaking this work, particularly in clinical enterprises that most acutely evidence the sociopolitical artifacts portending suboptimal health-care access and provision.

In the long history of cancer research, every generation is granted the role of defending the public’s health “in its hour of maximum danger” (19). *JNCI Cancer Spectrum* encourages evidence-based deliberation via a special issue dedicated to scholarship examining and intervening on the social determinants of health and cancer care. *JNCI Cancer Spectrum* solicits your address of the aforementioned intervention opportunities; we particularly welcome scholarship documenting and evaluating interventions impacting health and wellness determinants (eg, cancer prevention and survivorship interventions) or care determinants (eg, infrastructure improvements or care team innovation). We further encourage scholarships patients and community members co-author. We expect that co-authored manuscripts desribe efforts indicating inclusion, trust and trustworthiness, and accountability (20) necessary for patient-centered research on intervention development, implementation, and dissemination. We intend for this issue to evidence a “new alliance for progress” converting our “good words [eg, identifying detrimental social determinants] into good deeds [ie, intervening on detrimental social determinants]” (19). We hope this opportunity inspires patient, clinician, and community collaboration on care delivery innovation. *JNCI Cancer Spectrum* welcomes “the energy, the faith, [and] the devotion” (19) you bring to this endeavor. Our Journal is convinced the work published in this special issue will illuminate and invigorate cancer research and practice, locally and globally.

## Data Availability

No new data were generated or analyzed for this editorial.
